# Contemplating Health Economics, Coding and Reimbursement in Orthotics, Prosthetics and Pedorthics

**DOI:** 10.33137/cpoj.v4i2.36125

**Published:** 2021-09-21

**Authors:** MJ Highsmith, CM Fantini, DG Smith

**Affiliations:** 1 School of Physical Therapy & Rehabilitation Sciences, Morsani College of Medicine, University of South Florida, Tampa, Florida, USA.; 2 U.S. Department of Veterans Affairs, Rehabilitation & Prosthetics Services, Washington, USA.; 3 Department of Physical Medicine and Rehabilitation, Uniformed University of the Health Sciences, Bethesda, Maryland, USA.; 4 Department of Orthopaedics and Sports Medicine, University of Washington, Seattle, Washington, USA.

**Keywords:** Coding, Economics, Fee for Service, Orthotics, Prosthetics, Reimbursement

## Abstract

Reimbursement to U.S. healthcare service providers is largely transitioning from fee for service to fee for value for those clinicians who code using current procedural terminology and through their coding, describe their professional services. The Orthotic, Prosthetic and Pedorthic profession (O&P), currently codes using a system that describes the devices they evaluate for, fabricate, fit and maintain and their professional services are incorporated into their codes. These O&P codes, in contrast to those for other healthcare disciplines, are predominantly product based rather than service based, focusing on product features and function more than clinical service. This editorial manuscript provides a brief overview of the system the US O&P profession uses currently, particularly in the context of other healthcare professions transitioning to value based coding and reimbursement and culminates in a call to action for the profession to academically consider the strengths and weaknesses of the current system relative to alternative systems.

## ECONOMIC SCIENCE IN ORTHOTICS, PROSTHETICS AND PEDORTHICS

Health economics in Orthotic, Prosthetic and Pedorthics (O&P) may be thought of as maximizing O&P related rehabilitation for those using these services given limited available resources. A body of literature exists on the subject of healthcare economics in O&P. Included in this body of work is a State of the Science Conference Proceeding from the American Academy of Orthotists and Prosthetists (AAOP-O&P's professional association in the U.S.) which includes twelve articles.^1^ Contributions in this work include projects commissioned by the American Orthotic and Prosthetic Association (AOPA-O&P's trade association in the U.S.) such as a report comparing costs and outcomes in Medicare recipients receiving prosthetic care with those who did not receive prosthetic care and also comparing those of selected orthotic care.^2^ Additionally, building upon previously published clinical literature on microprocessor knee technologies for patients with transfemoral amputation which studied topics such as function, quality of life, safety and other clinical issues,^3^ AOPA commissioned a comprehensive economic analysis on the cost effectiveness of microprocessor knee systems compared to alternatives.^4^ This review concluded that MPK systems represent good, or potentially superior, value for money to comparable interventions in other healthcare sectors including total knee arthroplasty or implantable cardioverter defibrillators as cited examples.^4^

Among many positive benefits, the State of the Science Proceeding and health economic O&P literature referenced thus far succeeded in:

1. Raising awareness of health economic issues in O&P.
2. Pulling together O&P's body of knowledge in the health economic space.
3. Identifying conclusions, evidence statements, recommendations, strengths, weaknesses and knowledge gaps in O&P health economic science.
4. Providing foundational, economic methodologies that may be used in O&P research.
5. Initiating a call to action for the O&P profession's research community to work toward adding economic analyses to ongoing and future research.

The aforementioned work also led O&P thought leaders to share and learn from healthcare disciplines outside of O&P to examine future directions relating to payment (i.e. reimbursement) structure. The purposes of this editorial are to point out a collection of health economic work in O&P, increase awareness of reimbursement changes taking place outside of O&P and to serve as a catalyst for conversation within the O&P profession by proposing a call to action related to potential next steps in advancing O&P's coding and reimbursement conversation.

## REIMBURSEMENT CHANGES TAKING PLACE OUTSIDE OF O&P

In 1965, the U.S. Congress created Medicare under Title XVIII of the Social Security Act.^5^ In 1966, the American Medical Association(AMA), in concert with other specialty organizations, developed the iterative, Current Procedural Terminology (CPT) system. The CPT system was developed to provide standardized, uniform language for describing medical procedures and services and initially had no relationship with reimbursement.^5^ The AMA reviews and updates CPT codes annually. Healthcare innovation since development of the CPT code system has necessitated updates numerous times across the decades. The Health Insurance Portability and Accountability Act (HIPAA) of 1996, required the Department of Health and Human Services to name standards and code sets for electronic transactions of health information. This catalyzed further assessment of the CPT system, expanding beyond the reporting of procedures and services and into the reporting of pay for performance measures. The CPT coding system is the basis for service provider reimbursement when used with a relative value modifier.^5^

A detailed history of coding systems is beyond the scope of this manuscript but the abbreviated history presented serves to show that an interest in, and legislative action supporting early pay for performance concepts can clearly be observed as far back as HIPAA's passage in 1996.^5^ Legislative push away from fee for service (FFS) and toward fee for value (i.e. value over volume) has continued since then. For example, multiple medical specialists including urologists, gastroenterologists and orthopedists have begun chronicling the movement and emphasis of value over volume.^6–9^ More specifically, the Merit Based Incentive Payment System (MIPS) is also being discussed by specialist communities.^10^

Cost, specifically rising healthcare cost, has been central to this discussion since the origins of reimbursed care provision.^11^ While it is a core issue, cost represents merely half of the discussion as it relates to value.^12^ This is because mathematically, value is equal to the difference in two interventions in terms of outcome divided by cost.^6^ While mathematically, the value discussion seems straight forward, it is actually very complex and includes many other factors such as patient selection, outcomes, risk stratification, patient registry, guideline based practice, care coordination and others. Use of clinical guidelines and registries and other best practices offer insight on practice decisions and may serve to reduce variability.^6^ As mentioned previously, the sum of all of this can be quantitatively described in terms of value, cost and quality or outcome. In medicine however, other variables are also measured and evaluated. These can include length of stay, readmission and complication rates.^6^

## CONSIDERATIONS FOR POTENTIAL NEXT STEPS FOR O&P

The history of the development of the Healthcare Common Procedure Coding System (HCPCS) level II device L codes used by O&P to describe devices and services provided are documented elsewhere.^13^ Briefly, AOPA and Blue Cross and Blue Shield from South Carolina developed the template for the HCPCS L code system in the 1970's. It was piloted in 1979 and soon after, other insurance companies began following use of the system.^13^ The development of the associated fee schedule reimbursement methodology can be found in the U.S. Omnibus Reconciliation Act of 1987, which was implemented in 1989. The associated values were based on average payment amounts measured from 1986 to 1987 and are updated annually. Regulations for this methodology are in the U.S. Code of Federal Regulations (42 CFR 414.200). These coding and reimbursement methodologies have been associated with controversy by third party groups to government agencies who provide reimbursement based upon them and within the profession itself. It is important to note that in contrast to the CPT system, the L code system has not been maintained and updated with the same degree of modernization and applicability to current clinical O&P practice.

For instance, the Offices of the Inspector Generals for the U.S. Department of Health and Human Services and for the U.S. Department of Veterans Affairs have published numerous reports related to O&P. Some have been more clinically focused, exploring issues of population description, quality of care and other non-fiscal or less fiscally focused matters.^14,15^ However, the majority of these have been related to questionable or fraudulent procurement, acquisition, payment and purchasing,^16–21^ coding^22^ or billing^23^ related to O&P.

Additionally, editorial publications from members of the O&P community have also speculated as to whether or not the current and continued use of HCPCS L codes is a viable path forward.^13,24,25^ Among the issues cited in these publications are: delays and uncertainty in the coding application process and the seemingly diminishing success rate of receiving newly developed codes for newly developed technology. The latter issue is further being discussed as a potentially limiting factor in the investment and pursuit of research and development efforts to explore creation of new technologies. The DARPA/LUKE arm technology^26–28^ was cited in 2008 as an example of a government funded technological advancement that may not be assigned a code^25^ and as of this writing in 2021, the component remains uncoded. Additional challenges with the current L code system include incorporation of professional service fees into the device reimbursement. This limits recognition of the contributions of the credentialed O&P clinician as a professional member of the healthcare team and perpetuates the image that the O&P clinician may serve more as device provider. Restated, because the L-codes describing O&P interventions are included within durable medical equipment (i.e. fee for device), the prosthetist-orthotist may not necessarily be afforded or regarded with comparable professional standing relative to providers who describe their services using CPT codes (i.e. fee for service providers). To the authors' knowledge, the O&P profession has not published data on the practitioner's work and service as assessed by time, mental effort, judgment, technical skill, physical effort, amortized educational costs (both entry level and continuing education), psychological stress and other factors as other professions have.

Other issues cited include an inordinately lengthy list of codes relative perhaps to other professions. Some O&P professionals have suggested there may be interest to reduce the number of codes while others have suggested that more codes, allowing greater descriptive specificity are needed. The debate over the number of codes is further complicated when considering assertions of misapplication of codes potentially due to misunderstanding. This is an issue echoed in a recent OIG report.^22^ There are numerous other points of debate with the current coding system for O&P. To be clear, not all of the O&P community agrees that the L-code system is dysfunctional or in need of replacement. Some have indicated it is adequate, some have indicated revision may be in order and still others may be interested in an alternative system all-together.^13,24,25^

## CALL TO ACTION

This manuscript provided a brief overview of the L-code system's origins, controversies, strengths and weaknesses. However, the purpose was to challenge the profession to begin to contemplate next steps of coding and reimbursement against a backdrop where all of healthcare and medicine and reimbursement is clearly moving from fee for service to fee for value while O&P is currently in a different place entirely using a fee for device model. It would seem change is imminent but maybe not. It would seem timely for O&P to academically evaluate the strengths, weaknesses, opportunities, threats, merits and other considerations of:

- staying with the current coding and reimbursement system,
- revising and updating the current system,
- alternate systems such as:
  - fee for service
  - fee for value
  - a hybrid system

Further, perhaps there would also be value in studying the history and methods used to formulate the coding and reimbursement systems of other providers and specialists as well as those of other nations.^29–38^ This could assist in determining if elements of other models could benefit O&P or if other methods and models would serve the profession better in their entirety. Moreover, such an exercise could potentially be what is needed to affirm the current system is the best system and should remain in place. If however the O&P profession were to embark on the aforementioned discussion and conclude that the current system is not meeting the profession's needs, perhaps completely reimagining the current HCPCS L coding and reimbursement methodology may be in order. To do this, it may be useful to objectively study and quantify the value of O&P professional service using methodologies outlined and previously used by other professions. Moving toward a novel, RVU based coding and reimbursement model used by and known to work for other healthcare service providers who also work with devices, could improve the professional standing of the O&P clinician within the interdisciplinary healthcare team and personify O&P professionals as service providers.

In summary, the prior SSC^1^ was comprehensive and provided important professional awareness of and a call to action to engage more in economic science related to the O&P body of knowledge. The current call to action is in the context of legislative action driving all areas of healthcare toward value over volume. This is essentially driven by aligning incentives in such a way to prioritize the patient's interests, in terms of their outcomes above all other considerations. In this, it seems wise for professions to lead and be an active part of change or risk having legislators and payers make decisions on behalf of involved professions.

## DECLARATION OF CONFLICTING INTERESTS

The opinions and assertions expressed herein are those of the authors and do not necessarily reflect the official policy or position of any government or healthcare agency or academic institution to include the Uniformed Services University, the Department of Defense and/or the Department of Veterans Affairs. Dr. Douglas Smith received salary support through the Uniformed Services University of the Health Sciences under Award number HU00011920105 to the Henry M. Jackson Foundation for the Advancement of Military Medicine, Inc., and has been prepared in collaboration with the Center for Rehabilitation Sciences Research, Department of Physical Medicine & Rehabilitation, Uniformed Services University and the Department of Rehabilitation, Walter Reed National Military Medical Center.

## SOURCES OF SUPPORT

None.

## AUTHOR SCIENTIFIC BIOGRAPHY

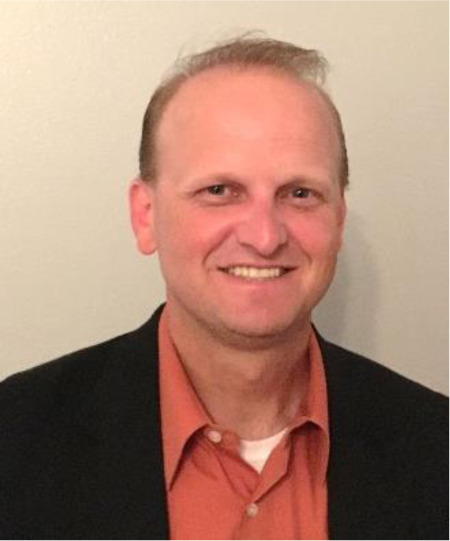



** M. Jason Highsmith**, PT, DPT, PhD, CP, FAAOP, attended Northwestern University's prosthetics program in 2004 and was certified following residency in 2006. He is dual licensed in physical therapy and prosthetics. He currently serves as the VA's National Director of Orthotic, Prosthetic & Pedorthic Clinical Services Program Office. He is joint appointed as a Professor at the University of South Florida (USF), is a Past-President of the American Academy of Orthotists & Prosthetists and is a Captain (Physical Therapist) in the US Army Reserves. Dr. Highsmith manages a considerable research portfolio and has published numerous peer-reviewed scientific manuscripts. The views expressed in this article are his alone, and do not represent the US Departments of Defense, Veterans Affairs, any university or any other organization.

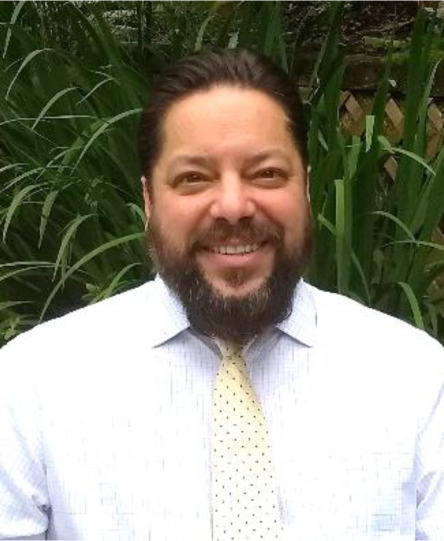



**Christopher Fantini**, MSPT, CP, BOCO, is a board-certified Prosthetist/Orthotist with over 25 years of physical rehabilitation experience. Mr. Fantini received a Master of Science in Physical Therapy from Columbia University in New York City and earned post graduate Certification in Prosthetics from Northwestern University in Chicago. Mr. Fantini has participated in notable work on advanced prosthetic devices to help facilitate the progression of emerging concepts & technologies to their clinical application. He has authored numerous peer reviewed articles as well as several chapters in texts relating to prosthetic care. He participates in the education/mentoring of Residents in the disciplines of Physical Medicine & Rehabilitation and Orthotics/Prosthetics. He has and continues to work on several projects relating to both direct patient care as well as strategic planning to advance the field of O&P. The views expressed in this article are his alone, and do not represent the US Department of Veterans Affairs or any other organization.

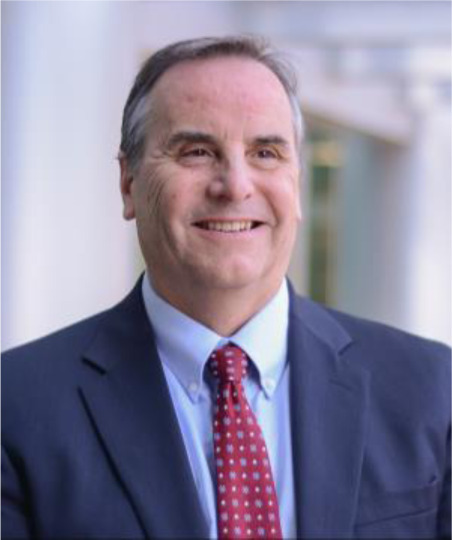



**Douglas G. Smith**, MD is a board-certified Orthopaedic Surgeon with over 30 years of experience in amputation surgery, the rehabilitation of individuals with limb loss, prosthetic prescription and prosthetic evaluation. He is Professor Emeritus in the Department of Orthopaedics and Sports Medicine at the University of Washington in Seattle. He is also a Professor in the Department of Physical Medicine and Rehabilitation at the Uniform Services University of the Health Sciences in Bethesda, Maryland. He works for the Henry M. Jackson Foundation for the Advancement of Military Medicine, and in his role as the Chief Orthopaedic Advisor for The Center for Rehabilitation Sciences Research in the department of Physical Medicine and Rehabilitation at The Uniform Services University he provides clinical education, consultation to the multi-disciplinary amputee care team and he supervises clinical research in limb salvage, amputation surgery and rehabilitation. He also serves on the American Orthotic and Prosthetic Association (AOPA) medical advisory board. The views expressed in this article are his alone, and do not represent the US Department of Defense, any university or any other organization.

## References

[1] Stevens PM, Highsmith MJ. An introduction to the proceedings of the AAOP'S state-of-the-science conference on the economic science of lower-limb prosthetic rehabilitation. J Prosthet Orthot. 2019;31(1S):1–2. DOI: 10.1097/JPO.0000000000000233

[2] Dobson A, El-Gamil A, Shimer M, DaVanzo JE. Economic value of prosthetic services among medicare beneficiaries: a claims-based retrospective cohort study. J Prosthet Orthot. 2019; 31(1S):94–100. DOI: 10.7205/MILMED-D-15-00545 26835740

[3] Highsmith MJ, Kahle JT, Bongiorni DR, Sutton BS, Groer S, Kaufman KR. Safety, energy efficiency, and cost efficacy of the C-Leg for transfemoral amputees: A review of the literature. Prosthet Orthot Int. 2010; 34(4):362–377. DOI: 10.3109/03093646.2010.520054 20969495

[4] Chen C, Hanson M, Chaturvedi R, Mattke S, Hillestad R, Liu HH. Economic benefits of microprocessor controlled prosthetic knees: a modeling study. J Prosthet Orthot. 2019; 31(1S):84–93. DOI: 10.1186/s12984-018-0405-8 PMC6157253 30255802

[5] Hirsch JA, Leslie-Mazwi TM, Nicola GN, Barr RM, Bello JA, Donovan WD, et al. Current procedural terminology; a primer. J Neurointerv Surg. 2015;7(4):309–312. DOI: 10.1136/neurintsurg-2014-011156 24589819

[6] Goldman AH, Kates S. Pay-for-performance in orthopedics: how we got here and where we are going. Curr Rev Musculoskelet Med. 2017;10(2):212–217. DOI: 10.1007/s12178-017-9404-9 28389971 PMC5435635

[7] Strazzabosco M, Allen JI, Teisberg EO. Value-based care in hepatology. Hepatology. 2017; 65(5):1749–1755. DOI: 10.1002/hep.29042 28073146

[8] Patel K, Presser E, George M, McClellan M. Shifting away from fee-for-service: alternative approaches to payment in gastroenterology. Clin Gastroenterol Hepatol. 2016; 14(4):497–506. DOI: 10.1016/j.cgh.2015.06.025 26122765

[9] Kaye DR, Miller DC, Ellimoottil C. Alternative payment models and urology. Curr Opin Urol. 2017; 27(4):360–365. DOI: 10.1097/MOU.0000000000000403 28441271 PMC5798610

[10] Hirsch JA, Rosenkrantz AB, Ansari SA, Manchikanti L, Nicola GN. MACRA 2.0: are you ready for MIPS? J Neurointerv Surg. 2017; 9(7):714–716. DOI: 10.1136/neurintsurg-2016-012845 27884928

[11] Lichtenstein RL. The United States' health care system: problems and solutions. Surv Ophthalmol. 1993; 38(3):310–316; discussion 316-317. DOI: 10.1016/0039-6257(93)90080-q 8310399

[12] Stevens PM, Highsmith MJ, Sutton BS. Measuring value in the provision of lower-limb prostheses. J Prosthet Orthot. 2019; 31(1S):23–31. DOI: 10.1097/JPO.0000000000000232

[13] Fairley M. L-Codes: Are They Meeting the Needs of O&P? [Internet]. The O&P Edge. 2008, [cited 2021, January 5], Available from: https://opedge.com/Articles/ViewArticle/2008-08-01/2008-08_01

[14] Daigh JD. Healthcare Inspection, Foot Care for Patients with Diabetes and Additional Risk Factors for Amputation [Internet]. U.S. Department of Veterans Affairs, Office of Inspector General. Report #11-00711-74. 2013, [cited 2021, January 5], Available from: https://www.va.gov/oig/pubs/VAOIG-11-00711-74.pdf

[15] Daigh JD. Healthcare Inspection. Prosthetic Limb Care in VA Facilities [Internet]. U.S. Department of Veterans Affairs, Office of Inspector General. Report #11-02138-116. 2012, [cited 2021, January 5], Available from: https://www.va.gov/oig/pubs/VAOIG-11-02138-116.pdf

[16] Jarmon GL. Medicare Improperly Paid Suppliers for Durable Medical Equipment, Prosthetics, Orthotics, and Supplies Provided to Beneficiaries During Inpatient Stays [Internet]. U.S. Department of Health and Human Services. Office of the Inspector General. A-09-17-03035. 2018, [cited 2021, January 5], Available from: https://oig.hhs.gov/oas/reports/region9/91703035.pdf

[17] Levinson DR. CMS has not Promulgated Regulations to Establish Payment Requirements for Prosthetics and Custom-Fabricated Orthotics [Internet]. U.S. Department of Health and Human Services. Office of the Inspector General. OEI-07-10-00410. 2012, [cited 2021, January 5], Available from: https://oig.hhs.gov/oei/reports/oei-07-10-00410.pdf

[18] Finn BJ. Audit of the Management and Acquisition of Prosthetic Limbs. U.S. Department of Veterans Affairs [Internet]. Office of Inspector General. Report #11-02254-102. 2012, [cited 2021, January 5], Available from: https://www.va.gov/oig/pubs/VAOIG-11-02254-102.pdf

[19] Brown JG. Medicare Losses Resulting from Early Payments for Durable Medical Equipment, Prosthetics, Orthotics and Supplies [Internet]. U.S. Department of Health and Human Services. Office of the Inspector General. OEI-03-99-00620. 2000, [cited 2021, January 5], Available from: https://oig.hhs.gov/oei/reports/oei-03-99-00620.pdf

[20] Brown JG. Medicare Payments for Orthotics. Inappropriate Payments [Internet]. U.S. Department of Health and Human Services. Office of the Inspector General. OEI-02-99-00120. 2000, [cited 2021, January 5], Available from: https://oig.hhs.gov/oei/reports/oei-02-99-00120.pdf

[21] Wright S. Use of Surety Bonds to Recover Overpayments made to DMEPOS Suppliers [Internet]. U.S. Department of Health and Human Services. Office of the Inspector General. OEI-03-11-00351. 1997, [cited 2021, January 5], Available from: https://oig.hhs.gov/oei/reports/oei-03-11-00351.pdf

[22] Reinkemeyer LM. Use of Not Otherwise Classified Codes for Prosthetic Limb Components [Internet]. U.S. Department of Veterans Affairs. Office of Inspector General. Report #16-01913-223. 2018, [cited 2021, January 5], Available from: https://www.va.gov/oig/pubs/VAOIG-16-01913-223.pdf

[23] Levinson DR. Questionable Billing by Suppliers of Lower Limb Prostheses [Internet]. U.S. Dept. of Health and Human Services. Office of the Inspector General. OEI-02-10-00170. 2011, [cited 2021, January 5], Available from: https://oig.hhs.gov/oei/reports/oei-02-10-00170.pdf

[24] Stark G, Stiner B. L-code obstacles: Experience from the front lines [Internet]. The O&P Edge. 2008. [cited 2021, January 5], Available from: https://opedge.com/Articles/ViewArticle/2008-08-01/2008-08_03

[25] Phillips Otto J. L-codes: What's wrong? What's right? [Internet]. The O&P Edge. 2008. [cited 2021, January 5], Available from: https://opedge.com/Articles/ViewArticle/2008-08-01/2008-08_02

[26] Resnik L, Latlief G, Klinger SL, Sasson N, Walters LS. Do users want to receive a DEKA Arm and why? Overall findings from the Veterans Affairs Study to optimize the DEKA Arm. Prosthet Orthot Int. 2014; 38(6):456–466. DOI: 10.1177/0309364613506914 24286806

[27] Resnik L, Klinger SL, Etter K. The DEKA arm: its features, functionality, and evolution during the veterans affairs study to optimize the DEKA arm. Prosthet Orthot Int. 2014; 38(6):492–504. DOI: 10.1177/0309364613506913 24150930

[28] Resnik L. Research update: VA study to optimize DEKA arm. J Rehabil Res Dev. 2010; 47(3):ix–x. DOI:10.1682/JRRD.2010.03.0034. 20665342

[29] Hsiao WC, Braun P, Becker ER, Dunn DL, Kelly N, Causino N, et al. Results and impacts of the resource-based relative value scale. Med Care. 1992;30(11 Suppl):NS61–79. DOI: 10.1097/00005650-199211001-00006 1434968

[30] Hsiao WC, Braun P, Dunn DL, Becker ER, Yntema D, Verrilli DK, et al. An overview of the development and refinement of the Resource-Based Relative Value Scale. The foundation for reform of U.S. physician payment. Med Care. 1992; 30(11 Suppl):NS1–12. DOI: 10.1097/00005650-199211001-00001 1434963

[31] Becker ER, Dunn D, Braun P, Hsiao WC. Refinement and expansion of the Harvard resource-based relative value scale: the second phase. Am J Public Health. 1990; 80(7):799–803. DOI: 10.2105/ajph.80.7.799 2356903 PMC1404989

[32] Hsiao WC, Becker ER. Paying physicians according to their resource-costs: the development of a resource-based relative value scale. Health Policy. 1989; 12(3):257–261. DOI: 10.1016/0168-8510(89)90075-4 10303776

[33] Hsiao WC, Braun P, Kelly NL, Becker ER. Results, potential effects, and implementation issues of the resource-based relative value scale. JAMA. 1988; 260(16):2429–2438. doi:10.1001/jama.1988.03410160105013 3050171

[34] Hsiao WC, Braun P, Dunn D, Becker ER. Resource-based relative values. An overview. JAMA. 1988; 260(16):2347–2353. doi:10.1001/jama.1988.03410160021004 3050169

[35] Hsiao WC, Braun P, Yntema D, Becker ER. Estimating physicians' work for a resource-based relative-value scale. N Engl J Med. 1988; 319(13):835–841. DOI: 10.1056/NEJM198809293191305 3412414

[36] Hsiao WC, Braun P, Dunn D, Becker ER, DeNicola M, Ketcham TR. Results and policy implications of the resource-based relative-value study. N Engl J Med. 1988; 319(13):881–888. DOI: 10.1056/NEJM198809293191330 3045557

[37] Hsiao WC. The resource-based relative value scale: an option for physician payment. Inquiry. 1987;24(4):360–361. 2961696

[38] Cutti AG, Lettieri E, Verni G. Health technology assessment as theoretical framework to assess lower-limb prosthetics—issues and opportunities from an international perspective. J Prosthet Orthot. 2019; 31(1S):55–73. DOI: 10.1097/JPO.0000000000000235

